# hucMSC-sEVs-Derived 14-3-3*ζ* Serves as a Bridge between YAP and Autophagy in Diabetic Kidney Disease

**DOI:** 10.1155/2022/3281896

**Published:** 2022-09-22

**Authors:** Siqi Yin, Wanzhu Liu, Cheng Ji, Yuan Zhu, Yunjie Shan, Zixuan Zhou, Wenya Chen, Leilei Zhang, Zixuan Sun, Wenqin Zhou, Hui Qian

**Affiliations:** ^1^Department of Emergency Medicine, The Affiliated People's Hospital of Jiangsu University, Zhenjiang 212002, Jiangsu, China; ^2^Key Laboratory of Laboratory Medicine of Jiangsu Province, Department of Laboratory Medicine, School of Medicine, Jiangsu University, Zhenjiang 212013, Jiangsu, China; ^3^NHC Key Laboratory of Medical Embryogenesis and Developmental Molecular Biology & Shanghai Key Laboratory of Embryo and Reproduction Engineering, Shanghai 200040, China

## Abstract

As nanoscale membranous vesicles, human umbilical cord mesenchymal stem cell-derived small extracellular vesicles (hucMSC-sEVs) have attracted extensive attention in the field of tissue regeneration. Under the premise that the mechanisms of hucMSC-sEVs on the treatment of diabetic kidney disease (DKD) have not been revealed clearly, we constructed DKD rat model with success. After tail vein injection, hucMSC-sEVs effectively reduced blood glucose, maintained body weight and improved renal function in DKD rats. Notably, we found that hucMSC-sEVs suppressed YAP expression in renal cortical regions. Further in vitro experiments, we confirmed that the expression of YAP in the nucleus of renal podocytes was increased, and the level of autophagy was inhibited in the high-glucose environment, which could be reversed by intervention with hucMSC-sEVs. We screened out the key protein 14-3-3*ζ*, which could not only promote YAP cytoplasmic retention instead of entering the nucleus, but also enhance the level of autophagy in the cytoplasm. Ultimately, excessive YAP protein was removed by autophagy, a classic way of protein degradation. In conclusion, our study provides new strategies for the prevention of DKD and proposes the possibility of hucMSC-sEVs becoming a new treatment for DKD in the future.

## 1. Introduction

Diabetic kidney disease (DKD) is one of the most serious microvascular complications of diabetes mellitus and the leading cause of death and disability in end-stage renal disease (ESRD) patients [1, 2]. DKD is associated with increased stromal expansion, which is characterized by diffuse or nodular expansion of the mesangium and diffuse thickening of the glomerular and tubular basement membranes [3]. Podocytes, attached to the outside of the glomerular basement membrane (GBM), and their structural and functional changes are extremely important in the development and progression of DKD, which are mainly manifested in abnormal podocyte hypertrophy, disappearance, and apoptosis [4–6]. Currently, the clinical treatment strategies for DKD are blood glucose control, renin-angiotensin system inhibitors combined with multidisciplinary treatment, supplemented by reasonable and moderate regular exercise [7], etc., whereas patients have long-term dependence on drugs and are prone to side effects such as hypoglycemia, hemolytic anemia, liver, and kidney damage. Thus, it is urgent to find new strategies for the treatment of DKD.

Stem cell therapy provides an alternative strategy for modulating complex disease processes by inhibiting pathogenic mechanisms and promoting tissue regeneration [8]. It has been demonstrated that mesenchymal stem cells (MSCs) could delay the progression of DKD by inhibiting the inflammatory response and reducing kidney damage [9–11]. In addition, MSC-conditioned medium has been confirmed that it could inhibit oxidative stress and alleviate renal fibrosis caused by DKD, which revealed that MSCs could act through paracrine mechanism [12, 13]. In recent years, small extracellular vesicles (sEVs), the main paracrine components of MSCs, have set off a research boom. As important carriers of intercellular information transfer, they can selectively encapsulate proteins, nucleic acids (miRNAs, lncRNAs, and circRNAs), lipids, and are potential targets for clinical treatment [14–16]. As we previously demonstrated, the human umbilical cord mesenchymal stem cell-derived small extracellular vesicles (hucMSC-sEVs) acted a part in repairing tissue injury, such as liver failure [17], skin damage [18, 19], acute kidney injury [20, 21], and unilateral ureteral obstruction (UUO)-induced renal fibrosis [22]. However, whether and how hucMSC-sEVs protect against DKD remains unclear.

The related studies have elucidated the functions of Hippo-YAP signaling in the maintenance of glomerular development and filtration barriers, podocyte homeostasis, renal epithelial damage, and renal fibrosis in DKD [23, 24]. Yes-associated protein (YAP), a multifunctional intracellular connexin and transcriptional coactivator, is the core effector of Hippo signaling pathway, which takes part in signal transduction and gene transcription regulation in cells [25]. Phosphorylation of a key serine (Ser 127) of YAP in mammals confines the protein to the cytoplasm and no longer takes effects in target gene expression, which is regulated by 14-3-3*ζ* protein [26]. YAP has been confirmed to be closely related to renal fibrosis [22, 27], cell apoptosis [28], and epithelial-mesenchymal transition [29]. Previous study by our team has confirmed that the hucMSC-sEVs-derived 14-3-3*ζ* coordinated the Wnt signaling pathway by regulating YAP during skin regeneration [30]. Nevertheless, the function of YAP in DKD needs further exploration.

14-3-3 proteins include a highly conserved family of proteins that are widely present in different eukaryotic cells [31]. 14-3-3 protein has various isoforms including *β*, *ε*, *η*, *γ*, *τ*, *σ*, and *ζ* [32], which is widely involved in the regulation of biological processes, such as protein trafficking, signal transduction, cell cycle, apoptosis, and autophagy [33, 34]. Autophagy is a cellular self-protection that keeps cells from damage by degrading organelles and proteins [35]. In cisplatin-induced AKI models, we have demonstrated that hucMSC-sEVs-14-3-3*ζ* enhanced autophagy levels and reduced cell apoptosis [21, 36]. Emerging evidence suggested that dysregulated autophagy may contribute to renal glomerular and tubulointerstitial lesions in DKD [37, 38]. For this reason, autophagy performs a key role in the progression of DKD. However, whether hucMSC-sEVs delivered 14-3-3*ζ* can induce the activation of autophagy to prevent renal injury in DKD is unclear.

This study aimed to investigate whether hucMSC-sEVs could effectively alleviate DKD and the underlying molecular mechanisms. On the one hand, our results confirmed that hucMSC-sEVs-delivered 14-3-3*ζ* induced phosphorylation at Ser 127 and cytoplasmic retention of YAP. On the other hand, it also has been verified that hucMSC-sEVs-14-3-3*ζ* could enhance autophagy in podocytes. Then it was surprisingly found that YAP, retained in the cytoplasm, was encapsulated into autophagosomes, which further promoted the degradation of YAP. Taken together, these findings underscore the importance of hucMSC-sEVs in DKD injury repair and provide a theoretical basis for the prevention and treatment of DKD.

## 2. Materials and Methods

### 2.1. Ethics

All the experimental protocols were approved by the Medical Ethics Committee and Ethics Committee for Experimental Animals of Jiangsu University (2020161).

### 2.2. Cell Culture

The isolation and characterization of hucMSCs is briefly introduced as follows [39]. Fresh human umbilical cord tissues were collected from the affiliated hospital of Jiangsu University and processed into 1-mm^3^ tissue blocks within 2 h. HucMSCs were cultured in minimal essential medium alpha (*α*-MEM) containing 10% fetal bovine serum (FBS, Gibco) at 37°C with 5% CO_2_. We subsequently expanded a large number of hucMSCs and collected the supernatant of the 3rd to 5th generation. Rat podocytes were purchased from American Type Culture Collection (ATCC) and maintained in low-glucose DMEM with 10% fetal bovine serum at 37°C with 5% CO_2_.

### 2.3. hucMSC-sEVs Purification and Characterization

HucMSC-sEVs were isolated and purified by differential ultracentrifugation [40]. The final content was resuspended in PBS and subsequently passed through a 0.22-*μ*m filter to remove bacteria. The hucMSC-sEVs were stored at -80°C for long-term use. The protein contents of the purified hucMSC-sEVs were detected by bicinchoninic acid (BCA) protein assay kit (Vazyme). The morphologies of hucMSC-sEVs were observed by TEM (FEI Tecnai 12, Philips). The sizes and concentrations of hucMSC-sEVs were detected by NTA (NanoSight, Amesbury). The expression of hucMSC-sEVs surface markers, including CD9, CD81, and TSG101, and the lack of Calnexin and Albumin expression were determined by western blot.

### 2.4. Diabetic Kidney Disease Rat Model

Aged 8 weeks male Sprague-Dawley (S-D) rats, weight within 200-250 g, were purchased from Charles River (Beijing, China). The rats were fed with a regular diet at an ambient temperature of 22-25°C for 5 days and then fed with 45% high fat diet (HFD) for 4 weeks. After being fasted for 12 h with free access to water, HFD fed rats were treated with STZ (35 mg/kg in 0.1 M citrate buffered saline, pH = 4.5) via tail vein injection [41]. Random fasting blood glucose (≥16.7 mmol/L) was measured to evaluate whether the rat models were successful. Eight weeks later, hucMSC-sEVs (10 mg/kg) were injected into DKD rats through tail vein intravenously. Normal rats were fed with normal diet as control. All rat models were harvested 24 weeks after STZ injection, and the kidney tissues were sacrificed for follow-up experiments.

### 2.5. hucMSC-sEVs Labeling and Internalization

According to the manufacturer's protocol, hucMSC-sEVs were labeled with fluorochrome Dil (Red, Thermo Fisher). Then hucMSC-sEVs in PBS were mixed with Dil in the dark at 37°C for 30 min. The labeled sEVs were washed with PBS and filtered through a 100-kDa-molecular-weight cut-off ultrafiltration membrane (Millipore) at 1000 g for 30 min to remove the unbound dye. PBS was used as a negative control. Podocytes (1 × 10^4^ per well) were seeded in 6-well plates and incubated with Dil-labeled hucMSC-sEVs at 37°C for 12 h. The cells were washed with PBS and fixed in 4% paraformaldehyde. Nucleus were counterstained with Hoechst 33342 (Sigma). A confocal microscope was used to acquire sequentially fluorescent images (Thermo Fisher Scientific).

### 2.6. Cytoplasm and Nuclear Fractionation

Cytoplasm and nuclear fractionation was performed according to the manufacturer's instructions (Vazyme). Cells were suspended in isolation buffer A mixed with protease inhibitors and rotated at 4°C for 1 min. After 12,000 g centrifugation at 4°C for 5 min, supernatant was collected containing the cytoplasm fraction. The remaining cell debris were then suspended in isolation buffer B mixed with protease inhibitors and rotated at 4°C for 1 min and repeated three times every 10 min. Cytoplasm and nuclear fractionation were detected by western blot.

### 2.7. Knockdown of 14-3-3*ζ* in hucMSCs

To target 14-3-3*ζ* genes silence, a lentiviral expression vector containing the 14-3-3*ζ* shRNA sequence (Sigma) was designed and Lenti-GFP-shRNA as negative control vector. The Lenti-14-3-3*ζ* shRNA vectors were generated by ligating the vector Tet-pLKO-puro with 14-3-3*ζ* shRNA oligonucleotides (FUBio). To obtain sequences information of 14-3-3*ζ* shRNA oligonucleotides, please browse Table S1 in the Supplementary Material. HucMSCs were transduced with the prepared lentivirus (Lenti-14-3-3*ζ* shRNA or Lenti-GFP shRNA) at the suitable dosage and selected with 1 *μ*g/mL of puromycin (Invitrogen) for 15 days. The efficiency of 14-3-3*ζ* knockdown was evaluated by western blot.

### 2.8. Western Blot

Total proteins from kidney tissues and cells were extracted in radioimmunoprecipitation assay (RIPA) buffer. Equal protein amounts of tissue or cell lysates were separated by sodium dodecyl sulfate-polyacrylamide gel electrophoresis (SDS-PAGE) at 12% or 15% and transferred to polyvinylidene fluoride (PVDF) membranes superior. After being blocked with 5% skim milk for 1 h, the membranes were incubated with primary antibodies and horseradish peroxidase (HRP)-conjugated secondary antibodies and then detected by using an enhanced chemiluminescent (ECL) substrate detection system. Primary antibodies were incubated overnight at 4°C. The HRP-conjugated goat antirabbit and goat antimouse secondary antibodies (CWBIO) were incubated 90 min at RT. For more antibodies information, all primary antibodies involved in this study are summarized in Table S2 in the Supplementary Material.

### 2.9. Cell Transfection and Structured Illumination Microscopy Assay

Podocytes were seeded at 6-well plates and cultured for 24 h and then transfected mRFP-GFP-LC3 adenovirus according to the manufacturer's protocol (Han Heng Biology). After treatment, the cells were washed with PBS and fixed with 4% paraformaldehyde. Finally, the cells were then stained with Hoechst33342 for nuclear staining. The images were acquired with a structured illumination microscopy (Nikon, SIM). The yellow puncta were autophagosomes, and the red puncta were autolysosomes.

### 2.10. Immunoprecipitation (IP)

The cell pellet was collected in Co-IP buffer (Pierce™), and then the supernatant was subjected to IP using the indicated primary antibodies at 4°C overnight. The lysate was centrifuged and incubated with 30 *μ*L protein A/G gel at 4°C for 4 h. The collected protein complexes were washed 4 times with Co-IP buffer and analyzed by western blot. Catalogue number is listed in Supplementary Table S3.

### 2.11. Immunofluorescence and Immunohistochemistry Analysis

Podocytes were fixed in 4% paraformaldehyde for 60 min, then treated with 0.1% Triton membrane breaker for 20 min. The kidney tissue slices were deparaffinized. The primary antibodies were incubated at 4°C overnight. After washing with PBS, cells were incubated with goat anti-Mouse IgG, Alexa Fluor 488 (Invitrogen) or goat antirabbit IgG, Alexa Fluor 555 (Invitrogen) at RT for 2 h. Nucleus were stained with Hoechst33342. Images were captured with a fluorescent microscope (Nikon) or a structured illumination microscopy (Nikon, SIM). The slides were visualized with a confocal microscope (DeltaVision Elite, GE).

### 2.12. QRT-PCR

Total RNA from podocytes was extracted with TRIzol reagent. cDNA was reversed according to the SuperScript™ II RT kit manufacturer's instructions (Vazyme). The qRT-PCR was used to detect the expression levels of the target genes. *β*-Actin was used as the endogenous control. The specific primers were produced by Sangon Biotech, and their products are shown in Supplementary Table S4.

### 2.13. Statistical Analysis

All data are expressed as mean ± standard deviation (SD) by using Prism software (GraphPad, San Diego). The statistically significant differences between different groups were assessed using one-way analysis of variance (ANOVA) followed by Turkey's posttest. *p* values were adjusted for multiple comparisons when appropriate. *p* < 0.05 was considered statistically significant.

## 3. Results

### 3.1. The Characteristics of hucMSCs and hucMSC-sEVs

**Figure 1:**
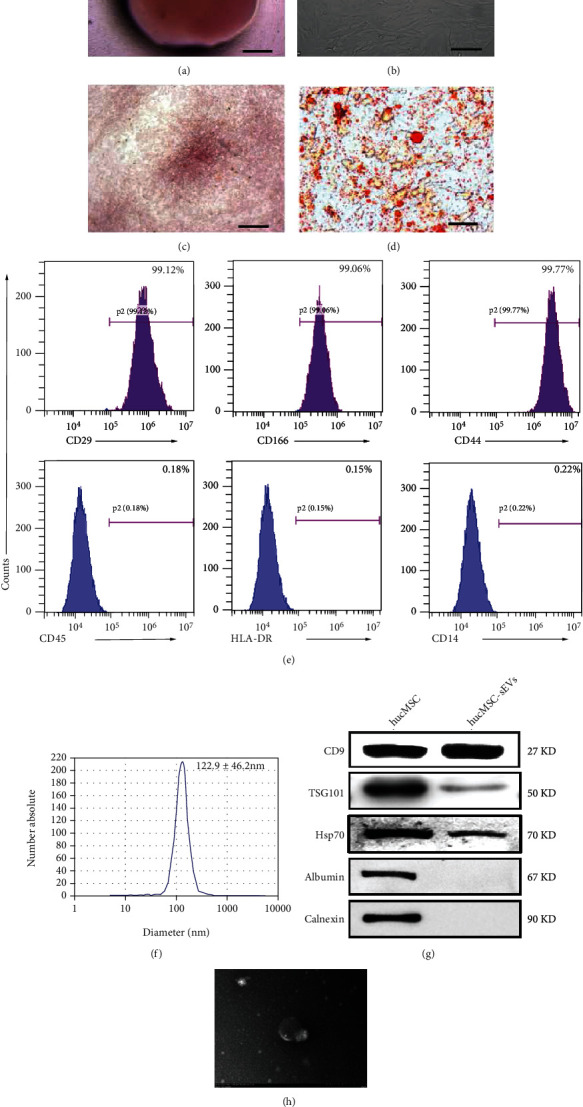
The characteristics of hucMSCs and hucMSC-sEVs. (a) Morphological identification of human umbilical cord tissue (40×, bar = 20 *μ*m). (b) The morphology of hucMSCs in 3rd generation was observed under microscope (100×, bar = 50 *μ*m). (c) Osteogenic differentiation of hucMSC was detected by neutrophil alkaline phosphatase (NAP) staining (100×, bar = 50 *μ*m). (d) Adipogenic differentiation of hucMSC was analyzed by Oil-Red-O staining (100×, bar = 50 *μ*m). (e) Flow cytometry analyses of phenotypic markers of hucMSC: CD29, CD166, and CD44. (f) Nanoparticle tracking analysis (NTA) was used to detect the average particle size and concentration of hucMSC-sEVs. (g) Detection of hucMSC-sEVs surface marker expression by western blot. (h) Representative TEM image of hucMSC-sEVs (bar = 200 nm).

Human umbilical cord tissues were purified and isolated as previously described. After 2 weeks, the hucMSCs were closely arranged around the umbilical cord tissue, which looked like long spindle-shaped and fish-like growth (Figures 1(a) and 1(b)). After cultured in osteogenic and adipogenic medium, Oil-Red-O staining revealed numerous lipid droplets in the hucMSC cytoplasm, and the cells became alkaline phosphatase positive (Figures 1(c) and 1(d)). These results showed that hucMSCs could be differentiated into both adipocytes and osteoblasts. Flow cytometry analysis demonstrated that hucMSCs highly express typical MSC surface markers, including CD29, CD166, and CD44, with the low expression of CD45, HLA-DR, and CD14 (Figure 1(e)). hucMSC-sEVs were isolated and purified from the cell culture supernatant by differential ultracentrifugation. Nanoparticle tracking analysis (NTA) indicated that hucMSC-sEVs were approximately 122.9 ± 46.2 nm in diameter (Figure 1(f)). Western blot assay was used to identify surface and interior markers expression such as CD9, TSG101, and Hsp70 except Calnexin and Albumin (Figure 1(g)). Transmission electron microscopy (TEM) revealed that hucMSC-sEVs displayed a classic bowl-shaped structure with a complete membrane (Figure 1(h)).

### 3.2. High-Expressed YAP Was Attenuated by hucMSC-sEVs in High-Glucose Environment

**Figure 2:**
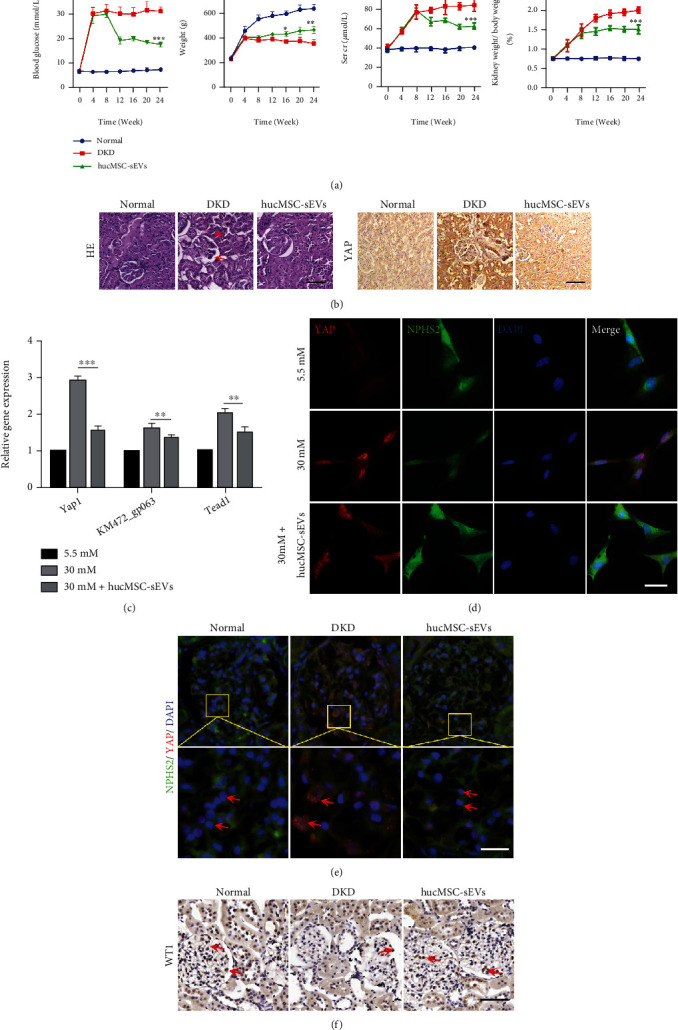
High-expressed YAP was attenuated by hucMSC-sEVs in high-glucose environment. (a) There were 3 groups: normal group, DKD group, and hucMSC-sEVs intervention group (i.v.). The blood glucose, body weight, creatinine, and blood urea nitrogen of the rats were counted weekly, and the statistical graph was drawn. (b) H-E staining and immunohistochemical detection of YAP expression in renal tissues at 24 weeks (bar = 100 *μ*m). (c) QRT-PCR detection of the expression level of Yap1, KM472_gp063, and Tead1 in podocytes. (d) The expression of YAP and NPHS2 in podocytes in high-glucose environment was observed by immunofluorescence method (bar = 25 *μ*m). (e) Tissue immunofluorescence analysis was used to observe the expression of YAP and NPHS2 in kidney tissue at 24 weeks (bar = 50 *μ*m). (f) Immunohistochemical staining showed the localization of WT1 (red arrows) in kidney tissue (bar = 100 *μ*m). (*n* = 3; ^∗^*p* < 0.05, ^∗∗^*p* < 0.01, and ^∗∗∗^*p* < 0.001).

After establishing a 24-week DKD rat model successfully, we injected hucMSC-sEVs at a dose of 10 mg/kg through the tail vein to observe the repair effect. Compared with normal and DKD groups, the intervention of hucMSC-sEVs effectively improved the symptoms of the DKD rats, which has reflected in a certain degree of hypoglycemic effect, maintaining a better body weight and reducing the levels of creatinine and urea nitrogen (Figure 2(a)). By means of hematoxylin-eosin staining (H-E staining) and YAP immunohistochemistry on renal tissue sections, pathological damage such as glomerular basement membrane thickening and renal tubular dilatation in DKD rats could be observed. The expression of YAP increased at the same time. hucMSC-sEVs inhibited the expression of YAP and alleviated renal tissue damage (Figure 2(b)). In vitro, first we stimulated podocytes with different gradients of glucose and found that 30 mM was the optimal concentration for high-glucose treatment (Supplementary Figure S1A-D). Immunofluorescence results of podocytes showed increased expression and entry of YAP into the nucleus, accompanied by decreased expression of the podocyte function-specific marker NPHS2, which were corrected after the intervention of hucMSC-sEVs (Figure 2(d)). Furthermore, treatment of podocytes in a high-glucose environment with insulin did not attenuate YAP expression nor inhibit YAP entry into the nucleus significantly, whereas hucMSC-sEVs did (Figure S1E-H). It was also confirmed from the mRNA level that YAP and its related transcription factors were highly expressed in high glucose-induced podocytes and hucMSC-sEVs could effectively attenuate their expression (Figure 2(c)). It was more intuitively observed by immunofluorescence that hucMSC-sEVs could effectively reduce the expression of YAP in the glomerular region of DKD rats, that is, hucMSC-sEVs could alleviate podocyte dysfunction caused by DKD. In addition, immunohistochemical staining showed that the number of cells positive for the podocyte nuclear marker WT1 was significantly decreased in the DKD group, while the number of podocytes increased after the hucMSC-sEVs intervention, which alleviated the podocyte loss that occurred during the DKD period (Figures 2(e) and 2(f)).

### 3.3. hucMSC-sEVs Reversed High Glucose-Induced Low-Level Autophagy in Podocytes

**Figure 3:**
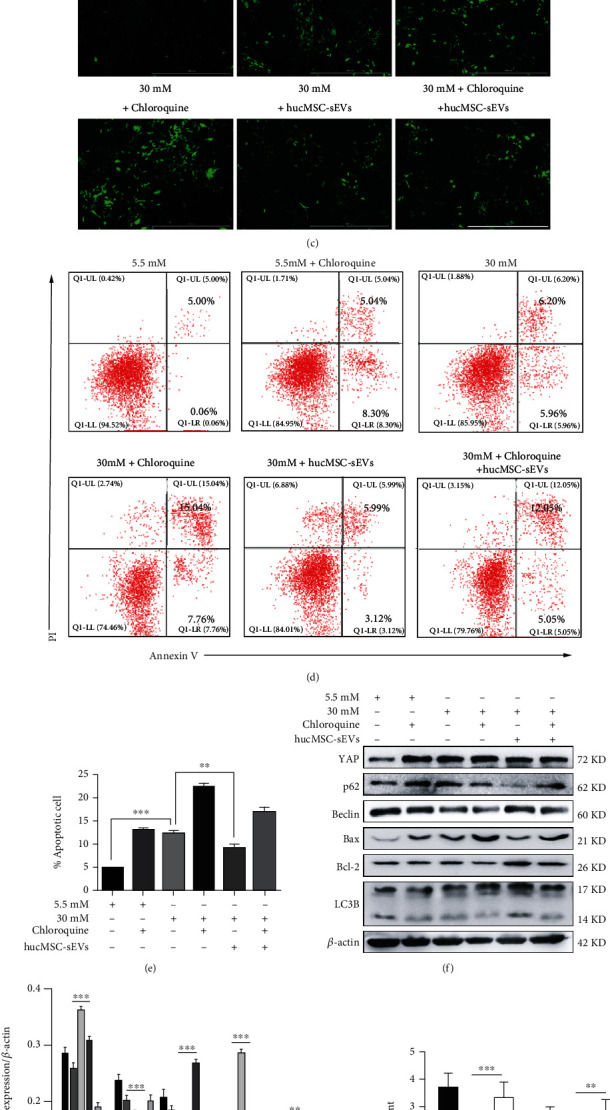
hucMSC-sEVs reversed high glucose-induced low-level autophagy in podocytes. (a) Flow cytometry analysis of autophagy-related proteins: LC3B, p62, and Beclin. (b) Statistical analysis of mean fluorescence intensity of autophagy-related proteins. (c) Transfected podocytes were visualized by fluorescence microscope to observe autophagy levels (bar = 1000 *μ*m). (d) Flow cytometry analysis was conducted to detect the apoptosis of podocytes under different treatment conditions. (e) Statistical analysis of the proportion of apoptotic cells. (f) The expression of autophagy and proliferation-apoptosis-related proteins were quantified by western blot (*n* = 3). (g) Statistical analysis of autophagy and proliferation-apoptosis-related proteins. (h) Quantification of podocyte autophagosomes and autolysosomes. (i) Representative TEM images of autophagosomes (red arrows) and autolysosomes (yellow arrows) in podocytes (*n* = 3; ^∗^*p* < 0.05, ^∗∗^*p* < 0.01, and ^∗∗∗^*p* < 0.001).

Flow cytometry analysis showed that under continuous high-glucose stimulation, the level of autophagy-related proteins (LC3B, p62, and Beclin) in podocytes were inhibited, which could be alleviated by hucMSC-sEVs (Figures 3(a) and 3(b)). After that, double-labeled LC3 (mRFP-GFP-LC3) lentivirus was transfected into podocytes for 48 h, and then high glucose and high glucose with hucMSC-sEVs were given. Subsequently, the intervention of chloroquine, an autophagy inhibitor, was used to further explore the changes in autophagy levels under different processing conditions. In the transfected podocytes, both the chloroquine group and the high-glucose treatment group appeared stronger GFP signal compared with the normal group, whereas the intervention of hucMSC-sEVs could effectively weaken the intensity of GFP signal (Figure 3(c)). The results of flow cytometry analysis showed that hucMSC-sEVs could mitigate the cell apoptosis of high glucose and/or chloroquine on podocytes (Figures 3(d) and 3(e)). The changes of autophagy-related proteins and proliferation-apoptosis-related proteins in podocytes were consistent with the above phenomenon (Figures 3(f) and 3(g)). Podocyte autophagosomes and autophagolysosomes were observed by transmission electron microscopy, and it was found that hucMSC-sEVs could promote the occurrence of autophagy (Figures 3(h) and 3(i)).

### 3.4. YAP Was Held in the Cytoplasm by hucMSC-sEVs-Derived 14-3-3*ζ*

**Figure 4:**
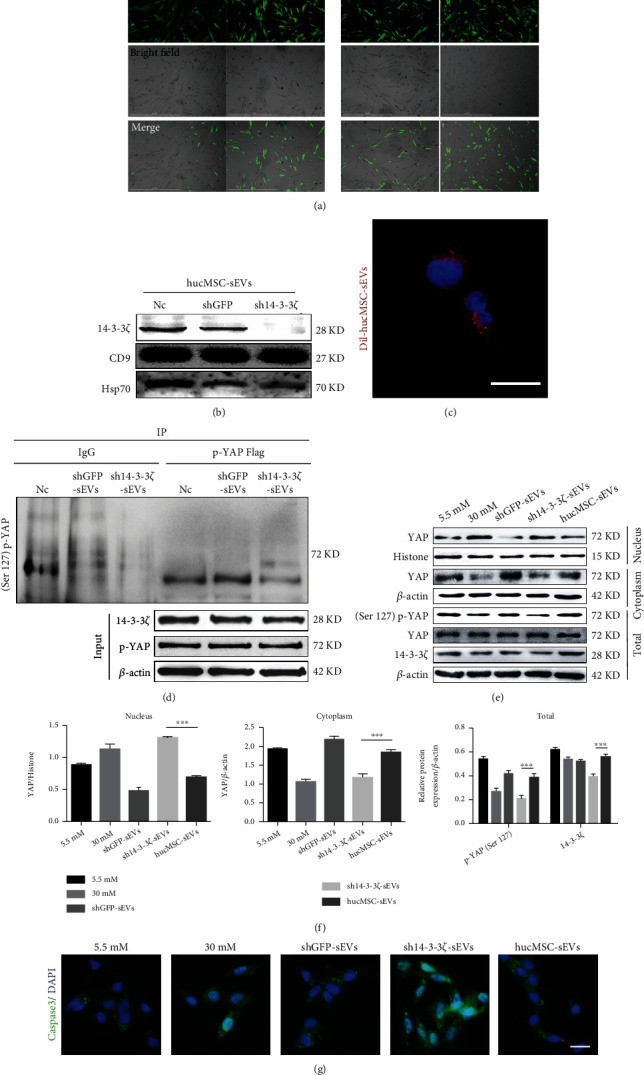
YAP was held in the cytoplasm by hucMSC-sEVs-derived 14-3-3*ζ*. (a) The lentiviral expression vectors containing the 14-3-3*ζ* shRNA sequence was designed and Lenti-GFP-shRNA as negative control vector. The lentiviral transfection efficiency was observed by fluorescence microscopy under different MOI values (bar = 1000 *μ*m). (b) Western blot experiments verified the knockdown efficiency of 14-3-3*ζ*. (c) The hucMSC-sEVs were labeled with fluorescent dye Dil and incubated with podocytes for 24 h. The podocytes were observed by confocal microscope to observe the endocytosis of sEVs (bar = 25 *μ*m). (d) Co-IP confirmed the binding of 14-3-3*ζ* to YAP phosphorylated at serine 127. (e) Nuclear-cytoplasmic separation assays were validated by western blot to measure cytoplasmic, nuclear, and total (p-)YAP levels (*n* = 3). (f) Statistical analysis of protein expression. (g) Cellular immunofluorescence was used to observe the apoptosis of podocytes (bar = 25 *μ*m). (*n* = 3; ^∗^*p* < 0.05, ^∗∗^*p* < 0.01, and ^∗∗∗^*p* < 0.001).

We screened the highly expressed 14-3-3*ζ* protein by LC-MS/MS analysis of hucMSC-sEVs. In order to verify the role of 14-3-3*ζ*, we constructed a 14-3-3*ζ* lentivirus stable transfection system for hucMSC cells (Figure 4(a)), and the knockdown efficiency was verified by protein surface labeling (Figure 4(b)). hucMSC-sEVs were labeled with Dil and uptake by podocytes (Figure 4(c)). Co-IP experiments confirmed that compared with the GFP control group, the expression of phosphorylated YAP at serine 127 in podocytes was reduced after the intervention of knockdown 14-3-3*ζ*-sEVs (Figure 4(d)). Further separation of nucleoplasmic proteins in podocytes verified that the nuclear translocation of YAP increased in a high-glucose environment. hucMSC-sEVs effectively inhibited YAP from entering the nucleus and blocking in the cytoplasm, which worked by 14-3-3*ζ* (Figures 4(e) and 4(f)). The apoptosis level of podocytes was clearly visualized by the fluorescence intensity and localization of Caspase3. The results confirmed that knockdown of 14-3-3*ζ* in hucMSC-sEVs could decrease the podocytes apoptosis to a certain extent (Figure 4(g)).

### 3.5. hucMSC-sEVs-14-3-3*ζ* Enhanced Autophagy to Engulf YAP

**Figure 5:**
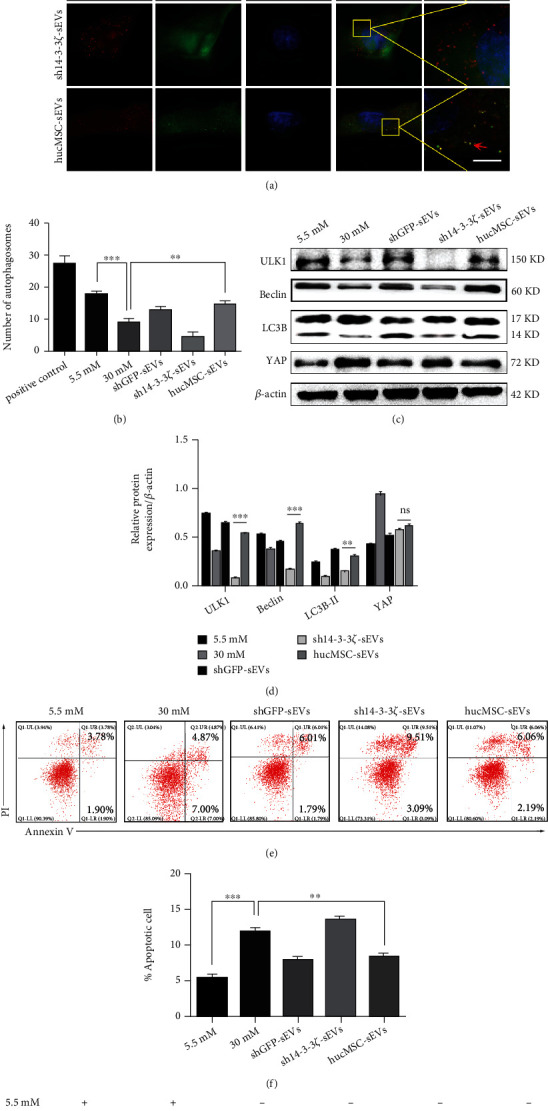
hucMSC-sEVs-derived 14-3-3*ζ* enhanced autophagy to engulf YAP. (a) The production and number of autophagosomes (marked as yellow dots) were observed by SIM (bar = 5 *μ*m). (b) Statistics of the number of autophagosomes in each group. (c) Compared with other control groups, the changes of autophagy-related proteins were detected by western blot (*n* = 3). (d) Statistical analysis of the protein expression. (e) Flow cytometry analysis was conducted to detect the apoptosis of podocytes under different treatment conditions. (f) Statistical analysis of the proportion of apoptotic cells. (g) Observation of the expression and colocalization of YAP and LC3B by SIM (marked as yellow dots) (bar = 5 *μ*m). (*n* = 3; ^∗^*p* < 0.05, ^∗∗^*p* < 0.01, and ^∗∗∗^*p* < 0.001).

After verifying the critical role of hucMSC-sEVs-14-3-3*ζ* on YAP, we still investigated the regulatory mechanism of 14-3-3*ζ* on autophagy. The structured illumination microscopy (SIM) was applied to observe the production of autophagosomes. The yellow puncta represented autophagosomes, and the red puncta were autolysosomes. The expression and the puncta of autophagosomes were decreased in the sh14-3-3*ζ*-sEVs group compared with the shGFP-sEVs group and the hucMSC-sEVs group (Figures 5(a) and 5(b)). Western blot results further confirmed that knockdown of 14-3-3*ζ* significantly suppressed the expression of autophagy-related proteins (Figures 5(c) and 5(d)). The number of apoptotic cells had significantly increased in sh14-3-3*ζ*-sEVs group compared with the control group, whereas pretreatment with hucMSC-sEVs resulted in fewer apoptotic cells (Figures 5(e) and 5(f)). Moreover, we unexpectedly found that the expression of YAP did not decline after the intervention of the autophagy inhibitor chloroquine (Figure 3(f)). Consequently, YAP was confirmed to colocalize with perinuclear LC3B in podocytes after chloroquine pretreatment (Figure 5(g)). It has been confirmed that YAP was enclosed in autophagosomes.

## 4. Discussion

Diabetic kidney disease (DKD) has become the leading cause of chronic kidney disease, placing a huge burden on the economy and society. The onset of DKD is insidious in the early stage, and the patients only pay attention to it when the symptoms of polydipsia and polyuria occur, so that it develops to advanced renal failure and even uremia. Due to the complex pathogenesis of DKD and the lack of specific and effective intervention targets, there is currently no breakthrough in treatment [42]. As far as the current clinical treatment of DKD is concerned, it is limited to only renin-angiotensin system inhibitors combined with multidisciplinary therapy [43]. Despite the addition of new clinical trials with renal outcomes as the primary endpoint, the progression of DKD has not been fully controlled. Therefore, it is urgent to find new treatment methods for DKD.

In the twenty-first century, stem cell therapy has opened the door to the medical field. Stem cells can be used as ideal “seed” cells for the repair of tissue and organ damage caused by pathological changes due to their high-efficiency proliferation, multidirectional differentiation potential, immune regulation, and self-replication [44]. Umbilical cord, adipose, and bone marrow-derived MSCs have been proved to have effects of inhibiting inflammation and antifibrosis and inhibiting apoptosis in DKD [45–49]. In addition, the paracrine approach of MSCs is considered to be more superior efficacy, among which are especially small extracellular vesicles (sEVs) [50]. sEVs, which encapsulate therapeutic proteins or RNA molecules, are not only promising biomarkers but also potential targets and tools for the treatment of DKD [51]. In this study, we successfully constructed high-glucose models both in vivo and in vitro, confirming that hucMSC-sEVs could effectively lower blood glucose level and reduce renal damage. This provides new ideas for the development of DKD treatment regimens.

YAP, a transcriptional regulator, has become the focus of great interest because of its remarkable biological properties in tissue organ development and tissue homeostasis. YAP activity is critical for cell proliferation throughout organ growth, tissue renewal, and regeneration [52, 53]. Previously, we confirmed that YAP is a key molecule in the treatment of renal fibrosis and the highly expressed YAP could be degraded by the CK1*δ*/*β*-TRCP ubiquitin system carried by hucMSC-sEVs in the UUO model [22]. In the DKD model of this study, we found that YAP was highly expressed in a high-glucose environment, accompanied by renal impairment and massive cell apoptosis both in vivo and in vitro. Afterwards, the key protein molecule, 14-3-3*ζ*, that played a vital role in hucMSC-sEVs were screened by LC-MS/MS technology. Via knocking down the 14-3-3*ζ* protein, the ability of hucMSC-sEVs to weaken YAP was reduced. Meanwhile, the apoptosis of podocytes was promoted at the same time. These evidences directly illustrate the importance of 14-3-3*ζ*.

The 14-3-3 protein family is widely distributed in eukaryotes and participates in a variety of signal transduction pathways and important cell life activities, such as cell growth and development, gene transcription, and cell apoptosis [54, 55]. According to studies, the 14-3-3 protein regulates the process of autophagy and the formation of autophagosomes by binding to autophagy-related proteins such as Beclin and hVPS34 [56]. Autophagy is a cellular process that determines cell fate and is tightly regulated by different signaling pathways, some of which, such as MAPK, PI3K and mTOR, are tightly regulated by the 14-3-3 protein [57]. Our previous related studies confirmed that 14-3-3*ζ* derived from hucMSC-sEVs could increase the level of autophagy and repair skin and acute kidney injury [19, 21]. However, in this study, we further confirmed that hucMSC-sEVs-derived 14-3-3*ζ* could increase the level of autophagy in podocytes and promote the formation of autophagosomes in DKD. Knockdown of 14-3-3*ζ* could attenuate the level of autophagy in podocytes, thus confirming the important role of 14-3-3*ζ* protein.

Autophagy is a highly conserved degradation system in which cellular contents such as proteins, organelles, and lipids are degraded in a lysosome-dependent manner. This may be related to the way in which p62-mediated ubiquitination-modified target proteins bind to autophagy receptors [58]. Lee et al. demonstrated that excess YAP is degraded by autophagy through the p62/Sqstm1-Nrf2 axis in HCC [59]. Additional studies have shown that enhanced autophagic activity in Alzheimer's disease promotes *β*-amyloid clearance in vitro and in vivo [60]. Here, we explored the “link” role of 14-3-3*ζ* protein in DKD. On the one hand, we first proved that the secretion of 14-3-3*ζ* protein from hucMSC-sEVs inhibited the entry of YAP into the nucleus and promoted its retention in the cytoplasm. On the other hand, we confirmed that 14-3-3*ζ* enhanced the level of autophagy in podocytes. In order to explore whether the highly expressed autophagy could remove excess YAP from the cytoplasm, the autophagy inhibitor chloroquine was used to block the formation of autophagic flux. It was found that chloroquine did not attenuate the expression of YAP, that is to say, the decrease of YAP was caused by the enhancement of autophagy. And after treated with chloroquine, YAP colocalized with perinuclear LC3B, which indicated that YAP was enclosed in autophagosomes.

**Figure 6:**
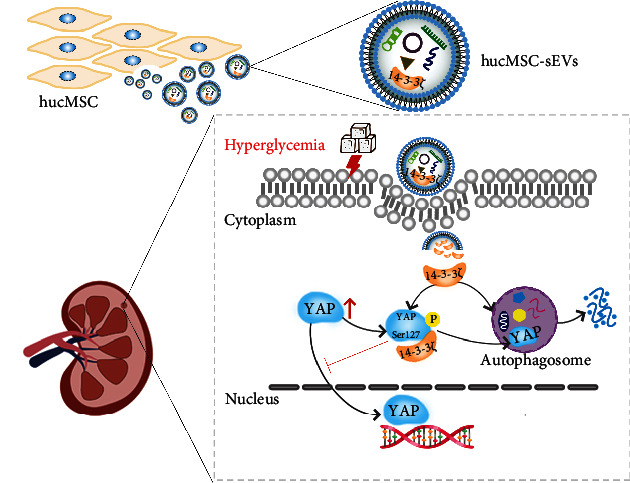
hucMSC-sEVs-derived 14-3-3*ζ* serves as a bridge between YAP and autophagy.

In conclusion, we demonstrate that YAP exacerbates renal damage and podocyte apoptosis in DKD. hucMSC-sEVs degraded cytoplasmic retained YAP in the way of increasing the level of autophagy by delivering 14-3-3*ζ*. This article demonstrated the potential of hucMSC-sEVs as nanoscale biomaterials for the treatment of DKD in the future (Figure 6).

## 5. Conclusion

Taken together, we demonstrated that the application of hucMSC-sEVs could reduce the apoptosis of renal podocytes induced by high glucose, inhibit the high expression of YAP, promote the activation of autophagy, and exert a cytoprotective effect. These results indicated that hucMSC-sEVs could inhibit YAP from entering the nucleus and linger in the cytoplasm by transporting 14-3-3*ζ* protein, which was eventually devoured and cleared by autophagy. Consequently, hucMSC-sEVs can be used as a new therapeutic tool for the prevention and treatment of DKD.

## Data Availability

The datasets of the current study are available from the corresponding author upon reasonable request.

## Additional Points

*Highlights*. (i) YAP protein was highly expressed in DKD both in vitro and in vivo, which was suppressed by hucMSC-sEVs. (ii) hucMSC-sEVs carried the internal 14-3-3*ζ* protein to promote YAP retention in the cytoplasm. (iii) hucMSC-sEVs-14-3-3*ζ* promoted a surge in the level of autophagy in podocytes. (iv) YAP, retained in the cytoplasm, was phagocytosed into autophagosomes.

## Supplementary Materials

Supplementary 1 Supplementary Figure 1 (Figure S1): merely lowering blood sugar did not prevent YAP overexpression in DKD.

Supplementary 2 Supplementary Table 1 (Table S1): the sequences of shRNA oligonucleotides. Knockdown 14-3-3*ζ* shRNA oligonucleotides, both forward and reverse, are displayed in Table S1.

Supplementary 3 Supplementary Table 2 (Table S2): the detailed information about antibodies in this study. The names, detection, manufacturers, and catalogue numbers of antibodies involved in this study are generalized in Table S2.

Supplementary 4 Supplementary Table 3 (Table S3): the names, manufacturers, and catalogue numbers of the kits used in this article are listed in Table S3.

Supplementary 5 Supplementary Table 4 (Table S4): the gene names, protein names, forward, and reverse sequences of qRT-PCR primers (5′-3′).
