# Supplementary material for: hucMSC-sEVs-Derived 14-3-3ζ Serves as a Bridge between YAP and Autophagy in Diabetic Kidney Disease
Source: Oxid Med Cell Longev. 2022 Sep 22;2022:3281896. doi: 10.1155/2022/3281896 (PMC9527117; doi:10.1155/2022/3281896)
Supplement: Supplementary 1 — Supplementary Figure 1 (Figure S1): merely lowering blood sugar did not prevent YAP overexpression in DKD. [file 3281896.f1.docx]

Supplementary Figure 1.

**Figure S1. Merely lowering blood sugar did not prevent YAP overexpression in DKD.**

**
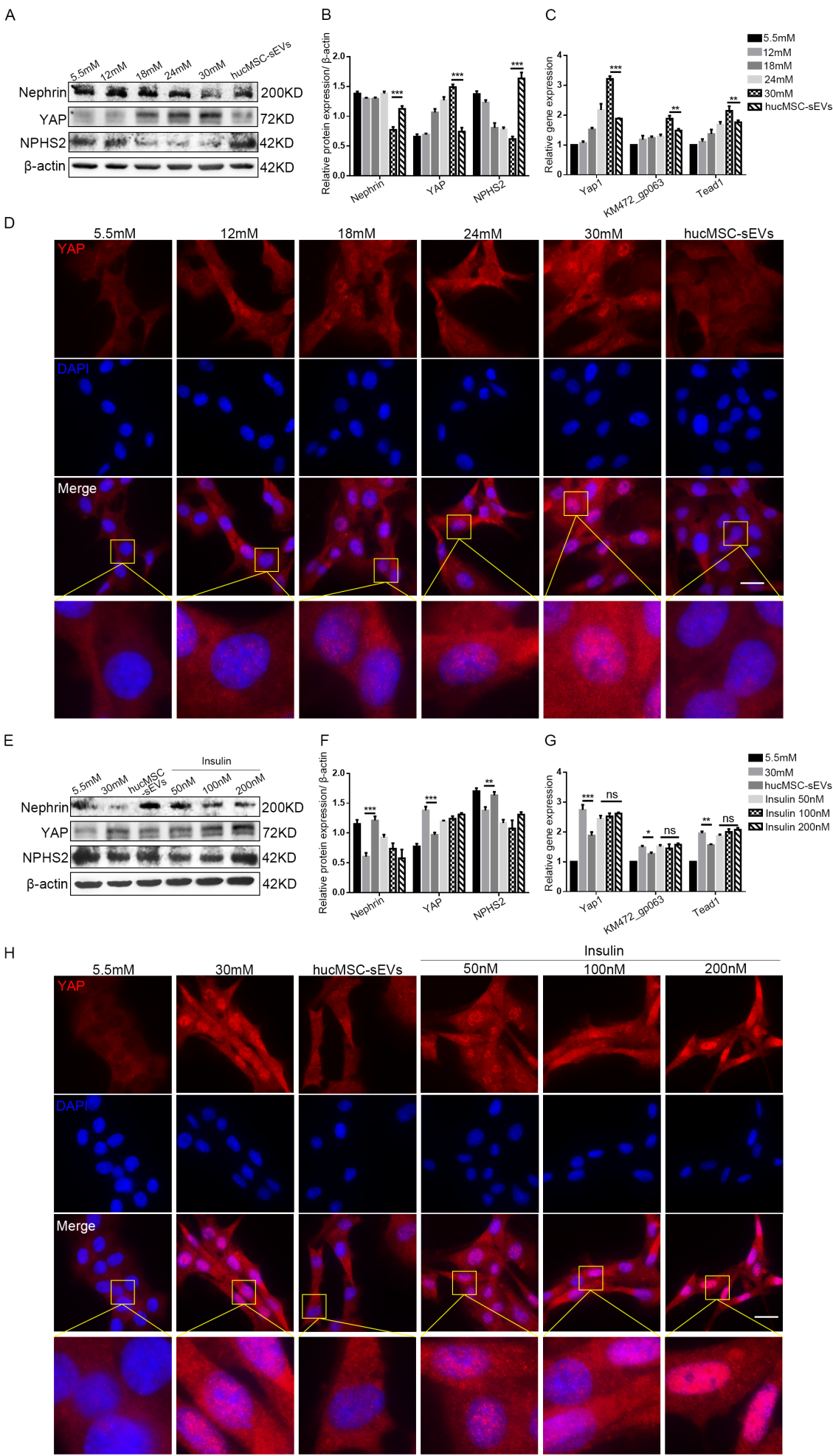
**

(A) Western blot assay was used to detect the protein expression levels of YAP, Nephrin and NPHS2 in podocytes under different concentrations of high glucose *(n=3)*. (B) Statistical analysis of protein expression. (C) QRT-PCR detection of the expression level of Yap1, KM472_gp063 and Tead1 in podocytes. (D) The changes and nuclear entry of YAP in podocytes were observed under different glucose concentrations under confocal microscope (Bar=100 μm). (E) High glucose-stimulated podocytes were treated with insulin according to different concentration gradients. The expression of YAP, Nephrin and NPHS2 were detected by Western blot *(n=3)*. (F) Statistical analysis of protein expression. (G) QRT-PCR detection of the expression level of Yap1, KM472_gp063 and Tead1 in podocytes. (H) The expression and nuclear localization of YAP were detected by immunofluorescence in podocytes treated with different concentrations of insulin (Bar=100 μm). (*n = 3; *p < 0.05, **p < 0.01, ***p < 0.001*).
