# Supplementary material for: hucMSC-sEVs-Derived 14-3-3ζ Serves as a Bridge between YAP and Autophagy in Diabetic Kidney Disease
Source: Oxid Med Cell Longev. 2022 Sep 22;2022:3281896. doi: 10.1155/2022/3281896 (PMC9527117; doi:10.1155/2022/3281896)
Supplement: Supplementary 2 — Supplementary Table 1 (Table S1): the sequences of shRNA oligonucleotides. Knockdown 14-3-3ζ shRNA oligonucleotides, both forward and reverse, are displayed in Table S1. [file 3281896.f2.docx]

Supplementary Table 1.

Table S1. The sequences of shRNA oligonucleotides.

| Target Gene | Sequences (5’-3’) |
| --- | --- |
| 14-3-3ζ | Forward CCGGGCAGAGAGCAAAGTCTTCTATCTCGAGATAGAAGACTTTGCTCTCTGCTTTTTG |
|  | Reverse AATTCAAAAAGCAGAGAGCAAAGTCTTCTATCTCGAGATAGAAGACTTTGCTCTCTGC |
