# Supplementary material for: hucMSC-sEVs-Derived 14-3-3ζ Serves as a Bridge between YAP and Autophagy in Diabetic Kidney Disease
Source: Oxid Med Cell Longev. 2022 Sep 22;2022:3281896. doi: 10.1155/2022/3281896 (PMC9527117; doi:10.1155/2022/3281896)
Supplement: Supplementary 3 — Supplementary Table 2 (Table S2): the detailed information about antibodies in this study. The names, detection, manufacturers, and catalogue numbers of antibodies involved in this study are generalized in Table S2. [file 3281896.f3.docx]

Supplementary Table 2.

Table S2. The detailed information about antibodies in this study.

| **Antibody** | **Detection** | **Manufacturer** | **Catalogue numbers** |
| --- | --- | --- | --- |
| CD9 | WB | Cell signaling technology | 13403s |
| TSG101 | WB | Abcam | ab125011 |
| Hsp70 | WB | Cell signaling technology | 4873S |
| Calnexin | WB | Cell signaling technology | 2679S |
| Albumin | WB | Cell signaling technology | 4929S |
| YAP | WB/IHC/IF | Cell signaling technology | 12395S |
| Phospho-YAP (Ser 127) | WB/IP | Cell signaling technology | 13008T |
| 14-3-3ζ | WB/IP | Abcam | ab51129-8 |
| PCNA | WB/IHC | Bioworld Technology | BS1289 |
| Bax | WB | Cell signaling technology | 2772S |
| Bcl2 | WB | Cell signaling technology | 15071S |
| p62 | WB/IF | proteintech | 18420-1-AP |
| Beclin | WB | proteintech | 11306-1-AP |
| LC3B | WB/IF | Sigma  Cell signaling technology | L7543  2775S |
| ULK1 | WB | Cell signaling technology | 8054S |
| β-actin | WB | Abclonal | AC026 |
| NPHS2 | WB/IF | proteintech | 20384-1-AP |
| Nephrin | WB | abcam | ab216341 |
| WT1 | IHC | abcam | ab89901 |
| Goat anti-Rabbit IgG, FITC conjugated | IF | SAB | L3202 |
| Cy3 Goat anti-mouse IgG | IF | Abclonal | AS008 |
| Goat anti-Rabbit IgG (H+L) Secondary Antibody, HRP conjugate | WB | Invitrogen | 31460 |
| Goat anti-Mouse IgG (H+L) Secondary Antibody, HRP conjugate | WB | Invitrogen | 31430 |
