# Supplementary material for: hucMSC-sEVs-Derived 14-3-3ζ Serves as a Bridge between YAP and Autophagy in Diabetic Kidney Disease
Source: Oxid Med Cell Longev. 2022 Sep 22;2022:3281896. doi: 10.1155/2022/3281896 (PMC9527117; doi:10.1155/2022/3281896)
Supplement: Supplementary 4 — Supplementary Table 3 (Table S3): the names, manufacturers, and catalogue numbers of the kits used in this article are listed in Table S3. [file 3281896.f4.docx]

Supplementary Table 3.

Table S3. The detailed information about all kits in this study.

| **Reagent kit** | **Manufacturer** | **Catalogue numbers** |
| --- | --- | --- |
| bicinchoninic acid (BCA) protein assay kit | Vazyme | E112-02 |
| Cytoplasm and nuclear fractionation kit | Vazyme | E211-02 |
| SuperScriptTM II RT kit | Vazyme | R111-02 |
| miRNA 1st Strand cDNA Synthesis Kit (by stem-loop) | Vazyme | MR101-02 |
| AceQ qpcr sybr green master mix | Vazyme | Q111 |
| Pierce™ Co-IP kit | Pierce™ | 26149 |
