# Supplementary material for: hucMSC-sEVs-Derived 14-3-3ζ Serves as a Bridge between YAP and Autophagy in Diabetic Kidney Disease
Source: Oxid Med Cell Longev. 2022 Sep 22;2022:3281896. doi: 10.1155/2022/3281896 (PMC9527117; doi:10.1155/2022/3281896)
Supplement: Supplementary 5 — Supplementary Table 4 (Table S4): the gene names, protein names, forward, and reverse sequences of qRT-PCR primers (5′-3′). [file 3281896.f5.docx]

Supplementary Table 4.

Table S4. The detailed sequences of qRT-PCR primers (5’-3’).

| Gene name | Protein name | Primer | Sequences (5’-3’) |
| --- | --- | --- | --- |
| Yap1 | YAP | Forward | CAGGATGGCGGGACTCAAAA |
|  |  | Reverse | CTGCTCATGCTTAGTCCGCT |
| KM472_gp063 | Cy61 | Forward | ACTTGACCAGGCTGGCACTC |
|  |  | Reverse | CCACAGCACCGTCAATACAT |
| Tead1 | TEAD | Forward | CATTACTCCTACCGCATCC |
|  |  | Reverse | CCTGTGTGTCTCTGTTGG |
| [Actb](https://www.ncbi.nlm.nih.gov/gene/81822) | β-actin | Forward | GACCTGTACGCCAACACAGT |
|  |  | Reverse | CTCAGGAGGAGCAATGATCT |
